# Ambient Heat and Sudden Infant Death: A Case-Crossover Study Spanning 30 Years in Montreal, Canada

**DOI:** 10.1289/ehp.1307960

**Published:** 2015-03-06

**Authors:** Nathalie Auger, William D. Fraser, Audrey Smargiassi, Tom Kosatsky

**Affiliations:** 1 Institut national de santé publique du Québec, Montreal, Quebec, Canada; 2 University of Montreal Hospital Research Centre, Montreal, Quebec, Canada; 3 Department of Social and Preventive Medicine, University of Montreal, Montreal, Quebec, Canada; 4 Department of Obstetrics and Gynecology, University of Sherbrooke, Sherbrooke, Quebec, Canada; 5 Department of Occupational and Environmental Health, University of Montreal, Montreal, Quebec, Canada; 6 British Columbia Centre for Disease Control, Vancouver, British Columbia, Canada

## Abstract

**Background:**

Climate change may lead to more severe and extreme heat waves in the future, but its potential impact on sudden infant death—a leading cause of infant mortality—is unclear.

**Objectives:**

We sought to determine whether risk of sudden infant death syndrome (SIDS) is elevated during hot weather.

**Methods:**

We undertook a case-crossover analysis of all sudden infant deaths during warm periods in metropolitan Montreal, Quebec, Canada, from 1981 through 2010. Our analysis included a total of 196 certified cases of SIDS, including 89 deaths at 1–2 months of age, and 94 at 3–12 months. We estimated associations between maximum outdoor temperatures and SIDS by comparing outdoor temperatures on the day of or day before a SIDS event with temperatures on control days during the same month, using cubic splines to model temperature and adjusting for relative humidity.

**Results:**

Maximum daily temperatures of ≥ 29°C on the same day were associated with 2.78 times greater odds of sudden infant death relative to 20°C (95% CI: 1.64, 4.70). The likelihood of sudden death increased steadily with higher temperature. Associations were stronger for infants 3–12 months of age than for infants 1–2 months of age, with odds ratios of 3.90 (95% CI: 1.87, 8.13) and 1.73 (95% CI: 0.80, 3.73), respectively, for 29°C compared with 20°C on the day of the event.

**Conclusions:**

High ambient temperature may be a novel risk factor for SIDS, especially at ≥ 3 months of age. Climate change and the higher temperatures that result may account for a potentially greater proportion of sudden infant deaths in the future.

**Citation:**

Auger N, Fraser WD, Smargiassi A, Kosatsky T. 2015. Ambient heat and sudden infant death: a case-crossover study spanning 30 years in Montreal, Canada. Environ Health Perspect 123:712–716; http://dx.doi.org/10.1289/ehp.1307960

## Introduction

Sudden infant death syndrome (SIDS) is a leading cause of death among infants 1–12 months of age, but its underlying risk factors are poorly understood (Kinney and Thach 2009; Moon et al. 2007). Despite rates that decreased following international campaigns to promote supine sleep positions in the 1990s, SIDS continues to be an important cause of postneonatal mortality in countries throughout Europe and the United States (Hauck and Tanabe 2008; Kinney and Thach 2009; Moon et al. 2007).

Evidence suggests that the underdeveloped thermoregulatory capacity of infants increases susceptibility to thermal stress and risk of sudden death (Guntheroth and Spiers 2001). Although numerous studies have shown that SIDS is associated with exposures such as overwrapping, bundling, and other behaviors linked with overheating (Guntheroth and Spiers 2001; Task Force on Sudden Infant Death Syndrome and Moon 2011), surprisingly little research documents whether outdoor heat is a risk factor. This is particularly concerning in light of climate change, which is expected to lead to more frequent and intense heat waves in the future (Intergovernmental Panel on Climate Change 2013). Several studies have reported that extreme heat is associated with higher mortality in the elderly (Baccini et al. 2008; Basagaña et al. 2011; Smargiassi et al. 2009), but the only studies that to our knowledge investigated a possible association with SIDS used ecologic methods and reported no associations with elevated temperature (Chang et al. 2013; Scheers-Masters et al. 2004). The paucity of research on high temperature using individual-level data on SIDS is concerning, considering that extreme ambient heat is a biologically plausible risk factor, and that more intense heat waves this century are imminent.

In response to the call for research on future impacts of climate change and extreme heat waves on the health of infants (Shea and American Academy of Pediatrics Committee on Environmental Health 2007), we sought to measure the association between high outdoor ambient temperature and SIDS in a large North American metropolitan center.

## Methods

*Study design and population*. We undertook a case-crossover analysis of all SIDS cases before 1 year of age in metropolitan Montreal, Quebec, Canada, from April through October for the years 1981–2010, the 30-year period available to us. Montreal has a continental climate with hot summers and cold winters. Deaths during November through March were not considered because high temperatures were not encountered. In addition, SIDS is common during winter (Mage 2005), and elevated thermal stress from excessive clothing in cold weather may inadvertently bias or mask associations with high outdoor temperatures (Ponsonby et al. 1992b). To increase statistical power, we did not exclude bridge months that reached relatively high maximum daily temperatures, including April (29.4°C) and October (26.6°C).

SIDS cases were identified in vital statistics records of the Quebec health ministry using *International Classification of Diseases, 9th Revision* (ICD-9) and *10th Revision* (ICD-10) codes for principal cause of death (798.0, R95.0). There were 196 cases of SIDS during the time span covered, and 736,916 live births. For comparison, there were 3,869 infant deaths overall during the same period, including 1,009 deaths after 27 days of age.

*Measures of exposure*. We hypothesized that high heat exposures could lead to SIDS, and therefore used the maximum outdoor temperature recorded on the same day and day before a SIDS day or control day as two main exposures. We used both days because the exact time of death was unknown, and we could not capture the exact temperature at the time of the event. Maximum temperatures the preceding day are certain to have occurred before death, but temperatures on the same day may have been reached only after death for a proportion of cases. To explore delayed impacts, we used the maximum temperature 2 days before a SIDS day or control day as a secondary exposure. Hourly temperature data were provided by Environment Canada by 24-hr block for the meteorological center situated approximately 20 km from Montreal’s core (Smargiassi et al. 2009). Maximum daily temperature was modeled as a continuous variable, and odds ratios were estimated relative to a referent value of 20°C. This referent was selected because 20°C is a comfortable temperature generally not associated with thermal stress in Montreal, and has been used in previous research (Auger et al. 2014; Smargiassi et al. 2009).

Humidity was considered a potential confounder of the association between temperature and SIDS. Humidity data were collected at the same meteorological center as temperature and were measured using mean daily percent relative humidity (continuous). We did not adjust for air pollution, a potential causal intermediate in the pathway between temperature and mortality, because this could bias estimates of the total impact of temperature on SIDS (Buckley et al. 2014).

*Statistical analysis*. We computed descriptive statistics and compared the proportion of SIDS deaths that occurred after days with maximum temperature ≥ 28°C with the proportion on the same days for all other causes.

We used the case-crossover design for its strength in assessing associations between transient exposures such as temperature and acute outcomes that are rare, such as SIDS (Maclure and Mittleman 2000). Case-crossover analysis is increasingly used to estimate impacts of temperature on mortality (Basagaña et al. 2011; Basu and Ostro 2008). In this design, each SIDS case serves as its own control, and the statistical analysis consists of comparing temperature exposure at the time of the event to temperatures during a short interval around the time of the case. Because each case is its own control, case-crossover designs inherently adjust for potential individual confounders that vary little over time, such as socioeconomic status, smoking, co-morbidities, and year of birth. In addition, this design accounts for potential bias from seasonal variation in conception and birth (Basso et al. 1995). Case-crossover studies are not ecologic, but use individual rather than aggregate data as the unit of analysis (Künzli and Tager 1997).

To select control days, we used an ambidirectional time-stratified approach where the referent period was the calendar month; we matched days on which SIDS occurred to control days consisting of the same weekdays of the month of death (Levy et al. 2001). If a death, for example, occurred on a Saturday in July 2000, control days comprised all remaining Saturdays in that month. Thus, any potential confounders that were stable during the month, such as socioeconomic status, were automatically adjusted for despite lack of data on such characteristics. We selected the same weekday as controls, thereby automatically adjusting for mortality that might vary by day of week. Because SIDS is rare, bias due to the use of control days that occur during the same month, but after the day of death, will be negligible (Lumley and Levy 2000).

We used conditional logistic regression to calculate odds ratios and 95% confidence intervals (CIs) for the association between maximum temperature of event days relative to temperatures of control days (each temperature variable was modeled separately), and included spline terms with knots at the 10th, 50th, and 90th percentiles (Durrleman and Simon 1989). We verified that use of knots located at the 5th, 50th, and 95th percentiles, and a greater quantity of knots, did not affect the shape of the curves (data not shown). All models were adjusted for relative humidity.

To estimate age-specific effects of temperature, we stratified SIDS cases into two postneonatal periods (1–2 vs. 3–12 months) for separate analyses, based on research suggesting increased susceptibility of infants older than 2 months to thermal stress (Fleming et al. 1992). In addition we performed a secondary analysis of associations at 3–6 months of age, but we did not have sufficient numbers of cases to estimate associations separately for SIDS deaths before 1 month of age (*n* = 13) or for 7–12 months of age (*n* = 14).

In sensitivity analyses, we ran models that excluded humidity, and examined associations for data restricted to summer months only (defined as June–August). We used the restricted cubic spline (RCS) macro in SAS version 9.2 (SAS Institute Inc., Cary, NC, USA) for statistical analyses (Heinzl and Kaider 1997). Data were anonymized, and the institutional review board of the University of Montreal Hospital Centre waived the requirement for formal ethics review.

## Results

Maximum temperature ranged from –1.5°C to 33.8°C on days before SIDS occurred. The proportion of SIDS deaths that occurred after a day with a maximum temperature ≥ 28°C (11.7%) was higher than the proportion of all other causes of death on the same days (9.3%; Table 1). The postneonatal period accounted for 93% of all SIDS, with 89 cases occurring at 1–2 months and 94 at 3–12 months. Most SIDS occurred between 1 and 4 months of age (*n* = 154; 78.6%).

**Table 1:** Distribution of infant deaths according to maximum temperature the preceding day, Montreal, April–October 1981–2010 [*n* (%)].

Cause of death	< 20°C	20–27.9°C	≥ 28°C	Total (*n*)
SIDS
All ages, < 1 year	99 (50.5)	74 (37.8)	23 (11.7)	196
1–2 months	40 (44.9)	36 (40.5)	13 (14.6)	89
3–12 months	51 (54.3)	25 (26.6)	18 (19.2)	94
Other causes, < 1 year	1,800 (49.0)	1,532 (41.7)	341 (9.3)	3,673
Total	1,899 (49.1)	1,606 (41.5)	364 (9.4)	3,869
Maximum daily temperature is expressed as a categorical variable for descriptive characteristics only (one-tenth of days between April and October had maximum temperatures ≥ 28°C).				

Maximum temperatures were on average higher the day before SIDS days than on control days for all months except April and September (Table 2). From June through August, the hottest months of the year, temperatures the day before SIDS events were 0.8–1.9°C higher than on control days. On the day of SIDS, temperatures on case days were 1.7–2.5°C higher than on control days. There was little difference in temperature patterns between the day before and the same day of a SIDS event in April, May, September, and October. Table 2 also illustrates the wide fluctuation in temperature experienced in Montreal, with maximum temperatures falling below 0°C in April and reaching up to 34°C in August.

**Table 2:** Monthly weather conditions of case and control days, Montreal, April–October 1981–2010.

Month	No. of SIDS	Mean maximum temperature [°C (range)]				Relative humidity (%)			
		Previous day		Same day		Previous day		Same day	
		Cases	Controls	Cases	Controls	Cases	Controls	Cases	Controls
April	29	9.4 (–1.5, 17.7)	10.5 (0.5, 26.1)	9.5 (1.0, 22.8)	10.4 (0.0, 27.4)	60.6	62.3	60.5	61.0
May	23	18.6 (10.1, 27.1)	18.0 (8.6, 30.0)	18.2 (10.0, 27.7)	18.0 (8.3, 26.0)	60.2	59.9	61.6	60.9
June	29	24.9 (13.0, 32.7)	23.0 (9.5, 32.6)	24.8 (11.3, 33.5)	22.4 (9.8, 33.4)	67.0	66.1	63.3	66.9
July	25	26.1 (17.2, 33.8)	25.3 (12.6, 33.4)	27.0 (17.8, 33.8)	25.3 (13.0, 33.4)	68.9	69.6	64.9	69.5
August	22	26.1 (19.3, 32.2)	25.2 (13.8, 34.3)	27.2 (17.8, 33.0)	24.7 (16.6, 32.3)	71.4	71.6	72.8	70.0
September	36	18.8 (10.1, 29.5)	19.9 (9.9, 28.8)	18.7 (10.6, 30.9)	19.5 (10.6, 32.3)	73.7	73.5	75.7	74.3
October	32	12.9 (2.8, 23.8)	12.4 (4.0, 24.3)	13.3 (3.0, 23.9)	12.7 (3.7, 26.0)	75.1	73.3	73.9	72.8

**Figure 1:**
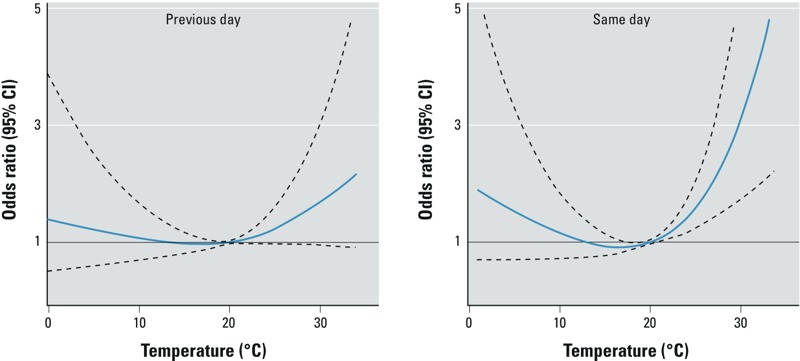
Association between maximum temperature and SIDS, Montreal, April–October 1981–2010. Odds ratio (solid blue line) and 95% CIs (dashed outer bands). All temperatures are relative to the 20°C mark, and are adjusted for mean relative humidity.

In spline models for all ages combined, the odds of SIDS increased steadily for same-day maximum daily temperatures > 20°C, with odds ratios of 1.41 (95% CI: 1.71, 1.69), 2.12 (95% CI: 1.43, 3.14), and 3.18 (95% CI: 1.76, 5.77) for 24, 27, and 30°C compared with 20°C, respectively (Figure 1). Associations with maximum temperatures on the previous day were weaker, with odds ratios of 1.18 (95% CI: 0.97, 1.42), 1.40 (95% CI: 0.96, 2.06), and 1.70 (95% CI: 0.94, 3.09) for 24, 27, and 30°C compared with 20°C, respectively. Temperature on the second day before death was not associated with SIDS (data not shown). There appeared to be a slight but statistically nonsignificant increase in the odds of SIDS with low temperatures on the day of death. At 5°C, for example, there was an odds ratio of 1.52 (95% CI: 0.70, 3.32) compared with 20°C.

**Figure 2:**
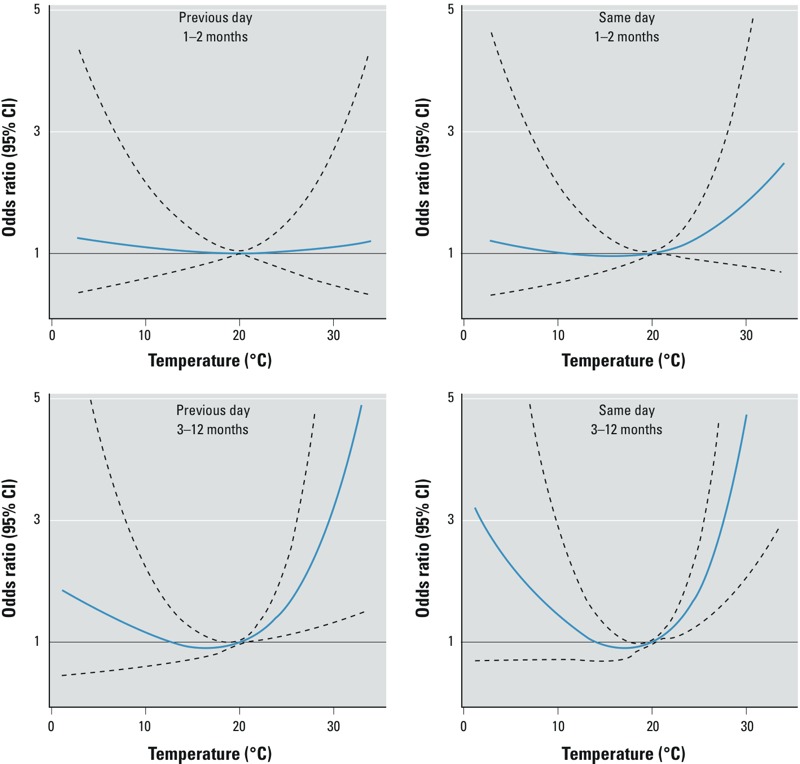
Association between maximum temperature and SIDS by postneonatal period, Montreal, April–October 1981–2010. Odds ratio (solid blue line) and 95% CIs (dashed outer bands). All temperatures are relative to the 20°C mark, and are adjusted for mean relative humidity. Associations for neonatal mortality were not computed because the number of cases was too low (*n *= 13).

When we ran spline models separately for the early and late postneonatal periods, the association between maximum temperature and SIDS was stronger for infants 3–12 months of age compared with those 1–2 months of age. Odds of SIDS in the 3–12 month postneonatal period were three to five times greater for temperatures ≥ 28°C relative to 20°C (Figure 2). This was the case for temperature both the day before and the day of death. Odds ratios at 30°C were 3.35 (95% CI: 1.33, 8.42) the day before and 5.03 (95% CI: 2.11, 11.96) the day of death compared with 20°C. In sensitivity analyses, restricting the analyses to SIDS from 3–6 months had no impact on the results (data not shown). The association with SIDS at 1–2 months was attenuated and statistically nonsignificant, although on the day of death the association was still positive with an odds ratio of 1.85 (95% CI: 0.78, 4.40) compared with an odds ratio of 1.12 (95% CI: 0.46, 2.74) the day before. Thus, the association between maximum temperature and SIDS both the day of and the day before was much stronger for SIDS at 3–12 months than at 1–2 months, suggesting that the findings for all ages combined were driven largely by cases that occurred at ≥ 3 months.

Change in location and quantity of knots had no impact on the shape of the spline curves (data not shown). Excluding relative humidity from models did not change the associations, and analyses restricted to summer months (June–August) removed the apparent increase in the odds of SIDS at very low temperatures without affecting associations at higher temperatures (results not shown).

## Discussion

*Main findings*. We found a strong association between elevated outdoor temperature and likelihood of SIDS on the day before and the day of death, specifically for infants ≥ 3 months of age. Thus, acute exposure to ambient heat may be a risk factor for SIDS mortality, especially after the first 2 months of life. To our knowledge, no previous research has investigated associations between high outdoor temperature and SIDS using methods that were not ecologic. This novel finding suggests that infants should be considered at increased risk of SIDS during hot weather, and that environmental alerts that include this group in health warnings may be warranted during heat waves. The findings also suggest that climate warming and the extreme heat events that are expected may contribute to a greater proportion of SIDS in the future.

*Comparison with other studies*. Most studies on temperature and risk of SIDS have focused on outdoor cold rather than heat (Campbell et al. 1991; Schluter et al. 1998; Williams et al. 1996). In four U.S. states, no association was found in an ecologic analysis of SIDS rates during a heat wave (Scheers-Masters et al. 2004). Although a Spearman correlation test suggested there was no association with temperature for 111 cases of SIDS, the rate of SIDS nonetheless appeared to increase progressively with the highest temperatures (Scheers-Masters et al. 2004).

Similarly, elevated maximum temperatures were not associated with 1,671 cases of SIDS in Taiwan (Chang et al. 2013). The investigators used an ecologic design relying on log-linear regression of 11 temperature categories with SIDS rates, but could not adjust for confounders. Another issue is that Taiwan has a mild climate with a population accustomed to heat, which may mitigate the impact of temperature on SIDS. A parallel may be seen in heat-related mortality among adults, which tends to be greater in northern countries, in spite of temperatures not as extreme as in the south (Anderson and Bell 2009; Curriero et al. 2002; Kalkstein and Davis 1989; McMichael et al. 2008). Lack of acclimatization is often evoked as a reason for the north–south mortality gradient in adults, and may also underlie the associations with SIDS in Montreal, a Canadian city just north of the U.S. border.

*Potential mechanisms linking extreme ambient heat with SIDS*. SIDS is hypothesized to result from a convergence of risk factors, including a pathologically vulnerable infant, a developmentally critical period, and exposure to external stressors that overwhelm autonomic functions (Kinney and Thach 2009; Paterson et al. 2006). Bedroom heating, prone or side sleeping, head covering, overwrapping, swaddling, and bed sharing are all associated with an increased risk of SIDS (Task Force on Sudden Infant Death Syndrome and Moon 2011). Although the pathophysiology behind these associations is unclear, these risk factors are all linked with thermal stress (Guntheroth and Spiers 2001), which may be exacerbated by brainstem abnormalities that impair thermoregulation (Kinney 2009). Indeed, high body temperatures have been found in cases of SIDS associated with prone sleeping (Ammari et al. 2009; Fleming et al. 1990; Ponsonby et al. 1993), head covering (Blair et al. 2008), bed sharing (Baddock et al. 2004), and overlayering (Fleming et al. 1990; Iyasu et al. 2002), particularly in heated rooms (Blair et al. 2008; Fleming et al. 1990; Ponsonby et al. 1993). These findings are also consistent with evidence that fans decrease risk of SIDS in warm room environments (Coleman-Phox et al. 2008). SIDS associated with overwrapping (Fleming et al. 1990) and night sweating (Kohlendorfer et al. 1998) is most common after 3 months of age, which aligns with our own results that suggested a stronger association with high temperature during the 3- to 12-month postneonatal period. Other factors, such as breastfeeding (Ip et al. 2007), may also contribute to age-related differences. Rates of breastfeeding decrease as infants age.

Heat stress is also thought to be behind seasonal patterns of SIDS, characterized by paradoxically higher rates in winter (Douglas et al. 1996; Jones et al. 1994; Kohlendorfer et al. 1998), presumably because infants are more heavily clad during cold weather and overheating is probable (Fleming et al. 1990; Ponsonby et al. 1992a). SIDS winter patterns may explain why studies have historically focused on associations with outdoor cold rather than heat (Campbell et al. 1991; Schluter et al. 1998; Williams et al. 1996). Our own results suggested an increase in the odds of SIDS at the lowest temperatures, although this finding should be interpreted with caution because we excluded cold months and precision was low. Nonetheless, our results align with the literature on cold temperature and SIDS (Campbell et al. 1991; Schluter et al. 1998; Williams et al. 1996), which supports our methodology and the associations we observed at high temperatures.

*Implications for future research*. We had measures of outdoor but not actual indoor temperatures. Indoor temperatures are correlated with outdoor weather, but may be affected by urban heat islands, building characteristics, and air conditioning. Consequently, homogeneous measures of outdoor temperature may nondifferentially misclassify actual exposures (Künzli and Tager 1997), potentially affecting the strength of the estimates to an unknown degree. Research on associations of SIDS with actual room temperatures is therefore merited. However, we used a time-stratified case-crossover design that naturally adjusts for confounders unlikely to vary significantly during any given month, such as usual sleep position, bed sharing, fan use, and air conditioning. Air conditioning can cause exposure misclassification, but should not be considered a confounder in this analysis because the case-crossover design by definition adjusts for individual characteristics. There is also the possibility that air conditioning prevented some SIDS from occurring, such that the cases in our sample occurred primarily in rooms that lacked air conditioning.

There may be merit in investigating alternate temperature or climate exposures. Evidence from a case-crossover study of 1,728 cases of SIDS in Shanghai suggests an association with wide diurnal temperature fluctuations, defined as the difference between maximum and minimum temperature during the day (Chu et al. 2011), suggesting that large changes in daily temperature may contribute to the risk of SIDS. Other indicators worth investigating include measures of apparent temperature that capture degree of comfort (Chung et al. 2009; Watts and Kalkstein 2004), or alternatively minimum temperatures (Kalkstein and Davis 1989). These and other novel climate exposures would be interesting topics for future research on SIDS.

Finally, generalizability of our findings to other areas, including the south where temperatures are higher and acclimatization greater, is to be determined. Larger-scale studies comparing associations across cities with different climates are warranted.

*Limitations of this study*. Limitations include a change in coding of cause of death from the 9th to the 10th revision of the ICD that occurred in 2000, although there is no evidence that SIDS rates were affected by this change in Canada (Gilbert et al. 2012). Moreover, coding changes would not likely vary by temperature. We excluded the months of November through March when high temperatures did not occur, so results for cooler temperatures should be interpreted with caution, especially because minimum (rather than maximum) temperatures may be more relevant. We could not explore potential modifiers of the impact of temperature on SIDS such as tobacco use, preterm birth, and maternal risk factors, though these individual-level risk factors cannot be confounders because they were naturally adjusted for in the case-crossover design.

## Conclusions

In this study, we found a strong association between high ambient temperature and SIDS at ≥ 3 months of age. Although more research is necessary to document associations in other settings, our study provides preliminary evidence to warrant limiting exposure of infants to high ambient temperatures. The American Academy of Pediatrics recommends avoiding overheating to prevent SIDS (Task Force on Sudden Infant Death Syndrome and Moon 2011), and our analysis provides support for including high ambient temperatures in this recommendation. Climate change will most likely result in more intense and frequent heat waves in the future, and the impact on risk of SIDS may not be benign. Potential effects of heat waves on SIDS need to be better understood, and thermoregulatory mechanisms involved in SIDS should be investigated more closely. Adaptation strategies for future climate warming, including medical alerts during heat waves, should include infants, a group that may be independently at risk of SIDS during periods of high ambient temperatures.
